# Elevated C-Reactive Protein Levels and Enhanced High Frequency Vasomotion in Patients with Ischemic Heart Disease during Brachial Flow-Mediated Dilation

**DOI:** 10.1371/journal.pone.0110013

**Published:** 2014-10-09

**Authors:** Shogo Watanabe, Eisuke Amiya, Masafumi Watanabe, Munenori Takata, Atsuko Ozeki, Aya Watanabe, Shuichi Kawarasaki, Tomoko Nakao, Yumiko Hosoya, Kohzo Nagata, Ryozo Nagai, Issei Komuro

**Affiliations:** 1 Department of Cardiovascular Medicine, Graduate School of Medicine, The University of Tokyo, Tokyo, Japan; 2 Department of Pathophysiological Laboratory Sciences, Nagoya University Graduate School of Medicine, Aichi, Japan; 3 Jichi Medical School, Tochigi, Japan; National University of Singapore, Singapore

## Abstract

**Purpose:**

The physiological role of vasomotion, rhythmic oscillations in vascular tone or diameter, and its underlying mechanisms are unknown. We investigated the characteristics of brachial artery vasomotion in patients with ischemic heart disease (IHD).

**Methods:**

We performed a retrospective study of 37 patients with IHD. Endothelial function was assessed using flow-mediated dilation (FMD), and power spectral analysis of brachial artery diameter oscillations during FMD was performed. Frequency-domain components were calculated by integrating the power spectrums in three frequency bands (in ms^2^) using the MemCalc (GMS, Tokyo, Japan): very-low frequency (VLF), 0.003–0.04 Hz; low frequency (LF), 0.04–0.15 Hz; and high frequency (HF), 0.15–0.4 Hz. Total spectral power (TP) was calculated as the sum of all frequency bands, and each spectral component was normalized against TP.

**Results:**

Data revealed that HF/TP closely correlated with FMD (r = −0.33, p = 0.04), whereas VLF/TP and LF/TP did not. We also explored the relationship between elevated C-reactive protein (CRP) levels and vasomotion. HF/TP was significantly increased in subjects with high CRP levels (CRP;>0.08 mg/dL) compared with subjects with low CRP levels (0.052±0.026 versus 0.035±0.022, p<0.05). The HF/TP value closely correlated with CRP (r = 0.24, p = 0.04), whereas the value of FMD did not (r = 0.023, p = 0.84). In addition, elevated CRP levels significantly increased the value of HF/TP after adjustment for FMD and blood pressure (β = 0.33, p<0.05).

**Conclusion:**

The HF component of brachial artery diameter oscillation during FMD measurement correlated well with FMD and increased in the presence of elevated CRP levels in subjects with IHD.

## Introduction

Vasomotion is oscillations in vascular tone or diameter that can be observed in many, if not all, vascular segments. Vasomotion is suggested to play a role in tissue oxygenation and other physiological responses. For example, changes in the pattern or amplitude of vasomotion can set the vascular resistance and conductance to the desired level [1]. However, the precise physiological role of vasomotion and its underlying mechanisms remain unclear. Mechanistic studies have suggested that vasomotion is generated by synchronous oscillations due to the release of calcium via gap junctions between adjacent smooth muscle cells [2]. Vasomotion is also regulated by various extra-myogenic factors including autonomic nervous system activity, hormonal factors, and endothelium-derived factors [2]–[5].

Rhythmic oscillations during microcirculation can be recorded in various ways depending on the site of observation. Oscillations in the resistance vessels can be recorded using photoplethysmography or near-infrared spectroscopy, whereas those in the skin nutritive and thermoregulatory vessels can be detected using laser Doppler flowmetry (LDF). Although vasomotion predominantly occurs during microcirculation [6], it can also occur in large muscular arteries [7].

The presence of low-grade chronic vascular inflammation is critical for the pathogenesis of atherosclerotic disease. Proinflammatory cytokines, such as tumor necrosis factor-α (TNF-α) and interleukin-6, have been implicated in the initiation and maintenance of the systemic and vascular inflammation that is associated with atherosclerosis [8]. Among the biomarkers of inflammation, C-reactive protein (CRP) is most potent prognostic factor in cardiovascular clinical application that is independently associated with the risk of incident or recurring cardiovascular events [9], [10]. These inflammatory factors also affect vascular function. For example, TNF-α and endotoxin impair endothelium-dependent vasodilation [11]. However, little is known about the relationship between vasomotion and inflammation exemplified by these inflammation parameters.

On the other hand, flow-mediated dilation (FMD), a response of vasodilation produced by increased blood flow shear, is a tool for evaluation of endothelial function. In subjects with ischemic heart disease (IHD), the value of FMD is generally impaired and more detailed vasomotion parameters is warranted for evaluating vascular function more minutely beyond FMD.

In this study, we observed oscillations in the brachial artery diameter during FMD in IHD, and we investigated the relationship between vasomotion of the brachial artery and several clinical variables, including C-reactive protein (CRP).

## Methods

### Subjects

We conducted a retrospective study of 37 patients with IHD who were hospitalized for cardiac catheterization. Inclusion criteria included coronary artery disease documented by angiography. Coronary artery disease was defined as the presence of at least one of the following:>50% luminal diameter narrowing of at least one epicardial coronary artery as shown by angiography, history of coronary revascularization, or history of myocardial infarction. Exclusion criteria included unstable clinical condition and significant valvular dysfunction. All components of standard informed consent, including the purpose of the study, risks, and benefits were fully explained to each subject, and written informed consent was obtained from all participants. The study protocol conformed to the Declaration of Helsinki and was reviewed and approved by the Institutional Review Board of the University of Tokyo (3003). All raw data was available in Table S1.

### Flow-mediated dilation (FMD) measurement and vasomotion analysis

Endothelial function was assessed using FMD, as described by the International Brachial Artery Reactivity Task Force [12]. The subjects were instructed to abstain from eating, smoking, and consuming caffeine for at least 4 h, and were asked to lie down for 20 min prior to the beginning of the study. FMD of the brachial artery was measured by amplitude-mode and brightness-mode ultrasonography using a linear-array 10-MHz transducer (UNEXEF18G, UNEX, Nagoya). After baseline diameter measurements were taken for 30 s, the blood pressure cuff was inflated to 50 mmHg above the patient's systolic blood pressure for 5 min, and then deflated. The diameter of the brachial artery was continuously recorded for 2 min after the cuff was deflated. All diameters were measured in the end-diastolic phase, which was defined as the beginning of R wave in the electrocardiogram. FMD was calculated as the percent change in the diameter from baseline before cuff release to the peak value after cuff release. The reproducibility of FMD was confirmed previously (*n* = 14) [13]. Intra-observer reliability yielded an intra-class correlation coefficient of 0.968, with variation of 3.2%.

We also performed spectral analysis of vascular diameter oscillations during FMD. These oscillations were analyzed using Maximum Entropy Calculation Methodology(MemCalc, GMS, Tokyo, Japan). Fourier Spectral analysis was performed on a brachial artery trace registered during the 2-min FMD measurements. Frequency-domain components were calculated by integrating the power spectrum in three frequency bands (in ms^2^): (1) very-low frequency (VLF), 0.003–0.04 Hz; (2) low frequency (LF), 0.04–0.15 Hz; and (3) high frequency (HF), 0.15–0.4 Hz. Total spectral power (TP) was calculated as the sum of all frequency bands.

### Heart rate variability (HRV)

Patients' electrocardiograms were also recorded during the FMD measurements using a three-lead electrocardiogram system, and the recorded data was converted to R–R intervals using the A/D converter within UNEXEF18G. HRV was analyzed in both the time and frequency domains of these recordings using the autoregressive method. Standard deviation of normal-to-normal beats (SDNN) was used as an indicator of both parasympathetic and sympathetic nerve activities, as mediated by baroreflex activity. SDNN was calculated from 100 heartbeats after cuff release during the FMD measurements. Frequency-domain components were calculated in a similar manner as vascular diameter oscillations. The power of LF and HF was calculated as the marker of sympathetic and parasympathetic nervous system activities.

### Blood samples

Blood samples from fasting subjects were collected for analysis within three days before or after FMD measurement. Hemoglobin and Hemoglobin A1c (HbA1c) levels, estimated glomerular filtration rate (eGFR), and low-density lipoprotein and CRP levels were measured using standard laboratory methods (Tokyo University Hospital).

### Statistics

Data are presented as mean±standard deviation. Differences between groups were calculated by Mann–Whitney test, t test and χ2 test. For quantitative variables that were not normally distributed, the Kendall rank correlation was calculated; otherwise, the Person correlation was calculated. A *p*-value of <0.05 was considered as statistically significant. Data analyses were performed using PASW Statistics 18 (SPSS Inc., Chicago) and JMP Pro 9 (SAS Institute, North Carolina).

## Results

### Subject characteristics

Table 1 lists the basic characteristics of the 37 subjects with IHD. A history of myocardial infarction was observed in 16.2 % of subjects. It also included 15 patients with diabetes mellitus, and 16 individuals were being treated with beta-blocking agents.

**Table 1:** Basic characteristics of the 37 IHD subjects.

Male/Female	25/12
Age (years)	67.7±7.5
Body mass index (kg/m^2^)	24.2±3.2
Systolic blood pressure (mmHg)	126.8±15.7
Diastolic blood pressure (mmHg)	65.5±11.3
Heart rate (Beat/min)	63.4±9.7
Hemoglobin (g/dl)	12.9±1.8
Hemoglobin A1C (%)	6.2±1.4
eGFR (ml/min/1.73 m^2^)	64.6±14.3
Low density lipoprotein (mg/dl)	95.7±29.5
CRP (mg/dl)	0.22±0.43
Brachial artery diameter (mm)	4.1±0.7
FMD (%)	4.2±1.5
SDNN (ms)	26.3±10.9
HRV LF/TP	0.24±0.14
HRV HF/TP	0.24±0.14
Smoker (%)	62.1
Myocardial infarction (%)	16.2
Diabetes mellitus (%)	40.5
Beta blocking agents (%)	43.2

IHD; ischemic heart disease, eGFR; estimated glomerular filtration rate, CRP; C-reactive protein, FMD; flow-mediated dilation; SDNN; standard deviation of normal to normal beats, HRV; heart rate variability, LF; low frequency, HF; high frequency, NS; not significant.

### Characteristics of Vasomotion

**Figure 1:**
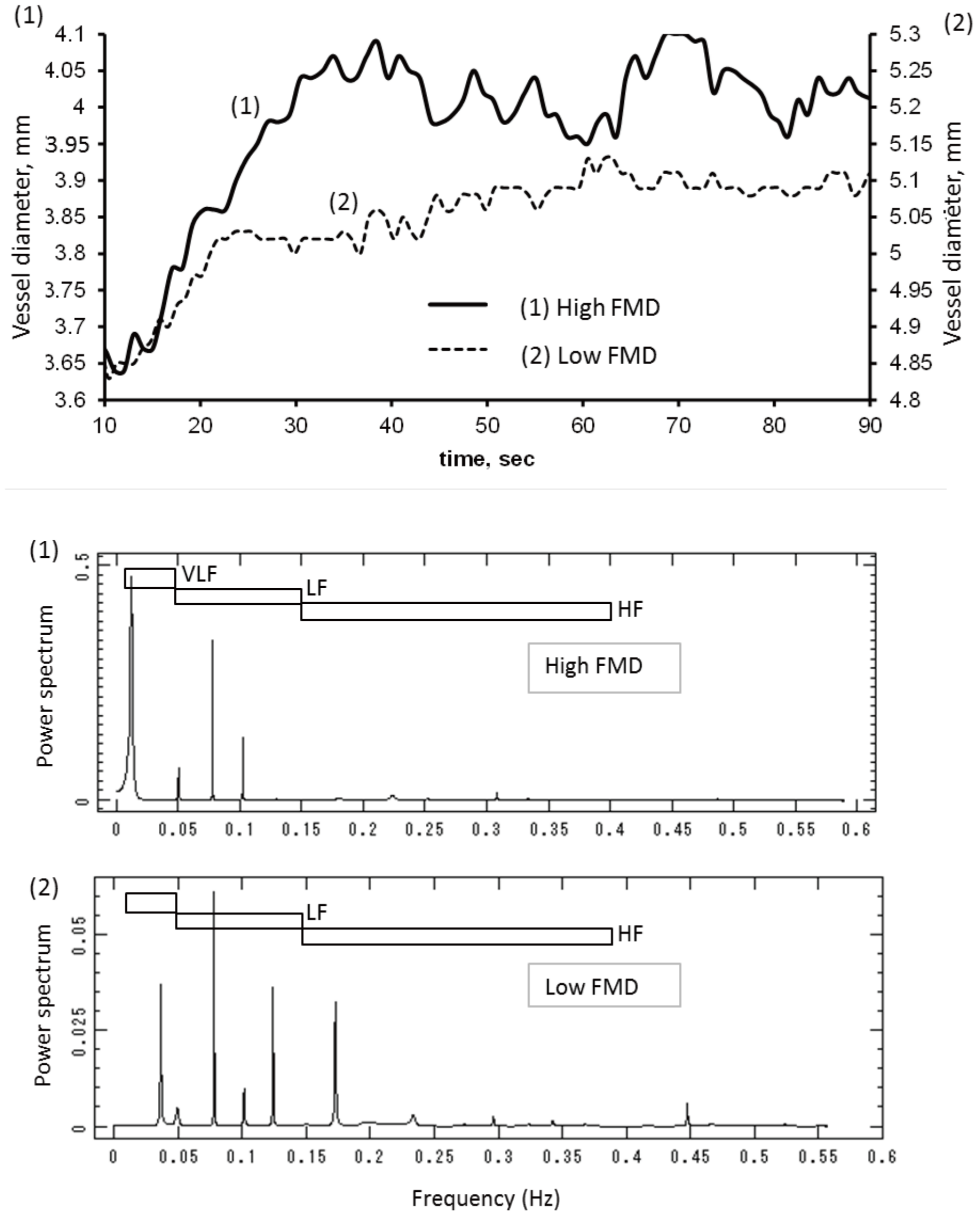
Examples of brachial artery vasomotion of a subject with high FMD (1) and low FMD (2). HF component was enhanced in this subject with low FMD. Brachial artery diameter was continuously measured after cuff release during FMD measurement. FMD, flow-mediated dilation; DM, diabetes; VLF, very-low frequency; LF, low frequency; HF, high frequency.

FMD and the spectral component of vasomotion were both measured as vascular parameters. Figure 1 shows an example of the time course of brachial artery dilation of a subject with high FMD and low FMD. The spectral components of vasomotion were sorted into three frequency bands: VLF, LF, and HF and the amplitude of each component was normalized by TP. The brachial artery generally began to dilate approximately 10–40 s after cuff release, and continued to dilate for 40–120 s. The time required for vasodilation (extended time) ranged from 30 to 80 s. The frequency spectrum of these changes in vascular diameter was estimated to be classified as VLF, whereas LF and HF components were estimated to reflect more minute oscillations. The spectral indices of the LF and HF components were 19±19% and 4.4±2.5% of TP, respectively (Table 2). When compared with the distribution of HRV, vasomotion of the brachial artery was more likely to fluctuate in the lower frequencies than heart rate oscillations. In particular, the HF component of vasomotion was significantly decreased compared with the LF component (Table 2).

**Table 2:** Comparison of HRV and vasomotion in subjects with IHD.

	HRV	Vasomotion
VLF/TP	0.52±0.18	0.74±0.18
LF/TP	0.24±0.14	0.19±0.19
HF/TP	0.24±0.14	0.044±0.025
LF/HF	1.7±1.9	5.1±4.2

HRV; heart rate variability, IHD; ischemic heart disease, VLF; very low frequency, TP; total power, LF; low frequency; HF; high frequency.

**Figure 2:**
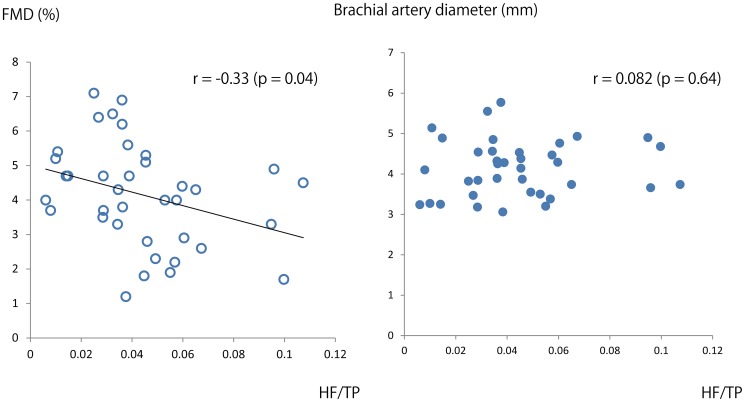
Scatter plot of the relationship between FMD or brachial artery diameter and the HF/TP power ratio of vasomotion in subjects with IHD. FMD, flow-mediated dilation; HF, high frequency; TP, total spectral power; IHD, ischemic heart disease.

Figure 1 also showed that the HF component of vasomotion variously changed among each subject. We investigated the relationships between the spectral components of vasomotion and clinical variables, including FMD, in subjects with IHD. Table 3 shows the correlation between the frequency components of vasomotion and each clinical variable. Data demonstrated that the HF/TP power ratio correlated with diastolic blood pressure (*r* = −0.46; *p* = 0.02), eGFR (*r* = −0.40; *p* = 0.02), CRP (*r* = 0.24; *p* = 0.04) and FMD (*r* = −0.33; *p* = 0.04), and HRV LF/HF (*r* = −0.37; *p* = 0.03) (Figure 2). The correlation between the HF/TP power ratio and these parameters suggested that the HF/TP component of vasomotion was influenced by factors in addition to endothelium-derived factors and autonomic nervous system activity. The lack of correlative relationship between brachial artery diameter and the HF/TP power ratio (*r* = 0.082; *p* = 0.64) suggested the correlation between FMD and the the HF/TP power ratio was independent from vessel diameter.

**Table 3:** Correlative relationship between clinical parameters and each frequency in vasomotion.

	HF/TP		LF/TP		VLF/TP		TP	
	r	p value	r	p value	r	p value	r	p value
Age (years)	0.18	0.28	0.16	0.34	−0.15	0.37	−0.12	0.47
Body mass index (kg/m^2^)	−0.088	0.60	0.051	0.76	−0.066	0.70	0.23	0.17
Systolic blood pressure (mmHg)	−0.29	0.08	−0.034	0.84	0.068	0.69	0.057	0.74
Diastolic blood pressure (mmHg)	−0.46	0.02*	−0.10	0.55	0.14	0.41	−0.14	0.40
Heart Rate (beat/min)	−0.12	0.55	0.14	0.41	−0.16	0.35	−0.083	0.63
Hemoglobin (g/dl)	−0.31	0.06	−0.082	0.63	0.098	0.56	−0.092	0.59
Hemoglobin A1C (%)	0.090	0.60	0.33	0.04*	−0.35	0.03*	0.098	0.56
eGFR (ml/min/1.73m^2^)	−0.40	0.02*	−0.13	0.43	0.22	0.20	0.34	0.04*
Low density lipoprotein (mg/ml)	−0.21	0.22	−0.20	0.25	0.26	0.12	0.14	0.42
CRP (mg/dl)	0.24	0.04*	−0.11	0.35	0.023	0.84	0.04	0.70
Brachial artery diameter (mm)	0.082	0.64	−0.21	0.22	0.16	0.36	0.062	0.71
FMD (%)	−0.33	0.04*	0.12	0.50	−0.080	0.64	−0.11	0.52
SDNN (ms)	−0.020	0.90	−0.12	0.47	0.15	0.37	0.023	0.89
HRV LF/TP	−0.15	0.37	−0.092	0.59	0.12	0.50	−0.034	0.84
HRV HF/TP	0.16	0.35	−0.033	0.85	0.031	0.85	−0.044	0.80
HRV LF/HF	−0.37	0.03*	−0.21	0.22	0.27	0.11	0.33	0.04*

VLF; very low frequency, TP; total power, LF; low frequency; HF; high frequency, Hb; hemoglonin, HbA1c; hemoglobin A1C; eGFR; estimated glomerular filtration rate, CRP; C-reactive protein, BAD; Brachial artery diameter, FMD; flow-mediated dilation; SDNN; standard deviation of normal to normal beats, NS; not significant, *; p<0.05.

We also investigated the contribution of the presence of the medication of beta-blocking agents on the behavior of vasomotion that affect the autonomic nervous system and the vascular tone. It unexpectedly revealed that there was no significant difference between subjects with and without beta-blocking agents in each component of oscillation (VLF/TP; 0.78±0.13 vs 0.70±0.21, LF/TP; 0.15±0.13 vs 0.23±0.22, HF/TP; 0.047±0.006 vs 0.041±0.006). Therefore, there was no significant effect of beta-blocking agents on the behavior of vasomotion.

### Effect of elevated CRP levels on vasomotion frequency bands

**Figure 3:**
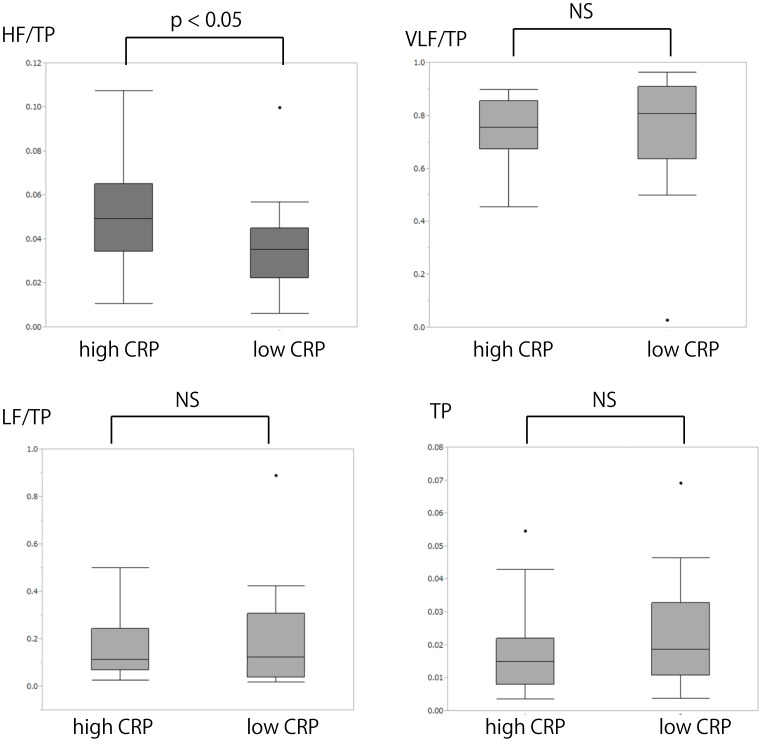
Comparison of each spectral component of vasomotion among subjects with high and low CPR level. HF, high frequency; TP, total spectral power; VLF, very low frequency; LF, low frequency; CRP, C-reactive protein.

We next explored the effect of elevated CRP levels on vasomotion. Subjects were divided into two groups based on their CRP level (median, 0.08 mg/dL). Each parameter of vasomotion was compared among subjects with high (>0.08 mg/dL) and low CRP (<0.08 mg/dL) levels. Clinical parameters including FMD and brachial artery diameter were not different between groups (Table 4). Blood pressure and heart rate were not also significantly different between these two groups because of the minute difference of CRP. We then investigated the relationship between the spectral component of vasomotion and elevated CRP levels (Figure 3). Of the different components of vasomotion (HF/TP, LF/TP, VLF/TP, and TP), HF/TP was significantly different between patients with high and low CRP levels (0.052±0.026 versus 0.035±0.022, respectively, *p*<0.05) suggesting that this was affected by the presence of elevated CRP levels. Consistent with this, there was a fine correlative relationship between HF/TP and CRP (*r* = 0.24, *p* = 0.04). By contrast, FMD was not correlated with CRP (*r* = 0.023, *p* = 0.84). In addition, elevated CRP levels significantly increased the value of HF/TP after adjustment for FMD and blood pressure (β = 0.33, p<0.05). This result suggests that HF/TP may reflect more minute influence of elevated CRP levels on vasomotion than the value of FMD.

**Table 4:** Characterization of subjects with high CRP and low CRP.

	High CRP	low CRP	
N	19	18	
Male/Female	14/5	11/7	NS
Age (years)	68.0±6.6	67.3±8.4	NS
Body mass index (kg/m^2^)	24.0±3.2	24.4±3.3	NS
Systolic blood pressure (mmHg)	127.2±18.0	126.4±13.3	NS
Diastolic blood pressure (mmHg)	64.5±9.7	66.7±12.9	NS
Heart rate (/min)	65.3±10.2	61.4±9.1	NS
Hemoglobin (g/dl)	12.5±1.9	13.3±1.7	NS
Hemoglobin A1C (%)	6.3±1.7	6.1±1.1	NS
eGFR (ml/min/1.73m^2^)	60.3±13.6	69.2±13.9	NS
CRP (mg/dl)	0.39±0.56	0.044±0.022	p<0.05
Brachial artery diameter (mm)	4.4±0.6	3.9±0.7	NS
FMD (%)	4.2±1.1	4.1±1.8	NS
SDNN (m)	24.0±8.6	28.6±12.7	NS
HRV LF/TP	0.22±0.13	0.25±0.17	NS
HRV HF/TP	0.27±0.15	0.21±0.12	NS
Smoker (%)	63.2	61.1	NS
Diabetes mellitus (%)	42.1	38.9	NS
Beta-blocker (%)	47.4	38.9	NS

BP; blood pressure, eGFR; estimated glomerular filtration rate, CRP; C-reactive protein, FMD; flow-mediated dilation; HRV; heart rate variability, LF; low frequency, HF; high frequency, NS; not significant.

## Discussion

To the best of our knowledge, this is the first study to investigate the oscillatory behavior of the brachial artery diameter. It correlated well with FMD and it can also detect the minute change of vasomotion in the presence of elevated CRP. The oscillating movements of the brachial artery have been recently documented [14], however it had not been clearly characterized. Oscillating movements measured in the brachial artery in this study are a direct measurement of vasomotion in blood vessels; therefore, this measurement might more accurately reflect the interaction among myogenic components, vessel diameter oscillations, and other interacting factors compared with vasomotor oscillations measured using other modalities.

### Spectral analysis of vasomotion

The mechanism of oscillatory calcium ion release in smooth muscle cells is responsible for vasomotion [15], [16]. However, these motions are greatly affected by extrinsic factors other than the smooth muscle cell system. In a study of LDF, VLF vasomotion was related to nitric oxide production by endothelial cells [17]. In contrast, LF vasomotion is considered to be under autonomic control. Decreased vasomotion around the LF spectral band in diabetic patients with sympathetic dysfunction, as demonstrated by Meyer et al., supports this hypothesis. [5]. These findings suggest the behavior of the vasomotion is modified by the extrinsic factors and that the source of oscillation may be predicted by the precise characterization of the oscillation spectrum.

In contrast, in the vasomotion of the brachial artery during FMD, the HF component was associated with several clinical parameters such as FMD, diastolic blood pressure and CRP, whereas the VLF or LF spectral components of vasomotion did not correlate with any clinical parameters except HbA1c. This character was markedly different from previous studies of vasomotion that were performed using other modalities [18]–[20]. Among factors that associated with HF component, FMD was a potent parameter; decreased FMD corresponded to an enhanced HF component of vasomotion. This suggests that the increase in the HF component of vasomotion reflects vascular impairment in endothelial function. In contrast, HRV LF/HF, another determining parameter, affected the HF component differently, because enhanced sympathetic nerve activity attenuated the HF component of vasomotion. In this way, the HF component of vasomotion was associated with endothelial function and autonomic nervous system activity.

The correlative relationship between decreased FMD and increased HF component suggests the increased HF component might be a compensatory mechanism that is triggered when tissue perfusion is compromised by vascular damage [15]. Podgoreanu et al. demonstrated that HF vasomotion emerged in the absence of physiological flow oscillation, and was derived from a peripheral origin through a parasympathetic nervous mechanism [21]. Our findings supported this enhancement of the HF component. In addition, the decrease of HF component with enhanced sympathetic nerve activity observed in the present study was compatible with the hypothesis that this compensatory mechanism is mediated through parasympathetic nervous system. However, further investigation is warranted to verify it.

### Elevated CRP levels and vasomotion

In this study, vasomotion also correlated with CRP. This correlation was independent of FMD and elevated CRP levels were shown to affect the HF component of vasomotion itself. There have been few reports that demonstrate the relationship between elevated CRP levels and the behavior of vasomotion. Schindler et al. demonstrated a direct association between systemic low-grade inflammation and vasomotor function [22], whereas Verma et al. reported that CRP levels did not correlate with the value of FMD [23]. The current study also failed to demonstrate an association between FMD and CRP. In contrast, the artery diameter oscillation measurement can detect the change derived from the presence of elevated CRP. Elevated CRP levels correlated with an increase in the HF component of vasomotion, suggesting that the inflammatory state, or specific humoral factors such as CRP or other inflammatory cytokines, might enhance the HF component in this subgroup of patients. Indeed, pro-inflammatory cytokines such as TNF-α activate calcium-related vasomotion by enhancing intracellular calcium levels and increasing the availability of calcium to refill the sarcoplasmic reticulum [24]. This enhanced calcium handling might lead to an increased HF component. CRP or other inflammatory cytokines also exert direct effects on the endothelium, which might also change smooth muscle vasomotion through endothelial modification [25]–[27].

### Physiological relevance and future directions

A vessel with an oscillating diameter has a higher conductance (and thus greater flow) than a vessel with a constant diameter of the same mean width [28]. In addition, oscillating contractions might be less costly in terms of energy use than constant contractions. Therefore, the increase of HF component in the presence of vascular injury is a reasonable physiological compensatory response to maintain the blood flow to peripheral tissue. In addition, the correlative relationship of the HF component with several clinical parameters suggested that the evaluation of the HF component could be a good risk parameter of vascular injury, which is difficult to detect using previous methods of vasomotion analysis.

We analyzed oscillating brachial arteries only during FMD. The analysis of vasomotion may disclose another aspect of vascular condition other than FMD. In this manner, the oscillating movements of the brachial artery in other conditions or changes in oscillation movements under different conditions might provide additional useful information regarding the conditions and characteristics of the measured vasculature. Therefore, this method can also be applied to other blood vessels such as the carotid artery. Examining the characteristics of vasomotion might be a promising tool during the diagnostic workup of cardiac patients.

## Conclusions

In the present study, we investigated the oscillatory behavior of the brachial artery diameter for the first time. The HF component of oscillation correlated well with FMD and increased in the presence of elevated CRP levels in subjects with IHD. Assessing vessel diameter oscillations in various clinical settings might allow a more accurate evaluation of the interaction between vasomotion and clinical conditions such as inflammation.

## Limitations

The primary limitations of this study were the small sample size and the retrospective nature of data collection. Because of the small sample size, there may possibly be some type II errors. For instance, other clinical parameters may be associated with the HF component of the brachial artery diameter oscillation or there may be association between FMD and CRP. In addition, performing multivariate analyses was limited because of small sample size in the current study. However, the relationships between elevated CRP levels and the enhancement of HF component remained significant after adjustment for FMD and blood pressure. The relationship between elevated CRP levels and the enhancement of HF component is sufficiently robust. Ideally, hypotheses should be tested in prospective studies with larger samples. We selected the median (0.08 mg/dl) for dividing subjects into groups with low and high CRP levels. There is no consensus about the threshold level of CRP level for predicting the risk of atherosclerosis. A similar value was applied in previous reports [23], [29], suggesting the value in the current study is reasonable. In addition, the interpretation of total autonomic nervous system activity might be limited by the fact that autonomic nervous system activity measurements were made solely during resting short-term HRV. Generally, vasomotion is reported to be a central feature to the microcirculation. However, the relationship between vasomotion observed in this study and that in the microcirculation remained to be unknown and further exploration is needed. Nitroglycerin-mediated dilation measurements were not performed because it was not possible to use nitroglycerin in all IHD subjects owing to the associated risks.

## Supporting Information

Table S1 **All raw data in this study.**
(XLSX)
